# Variations in cognitive abilities across the life course: Cross-sectional evidence from *Understanding Society*: The UK Household Longitudinal Study

**DOI:** 10.1016/j.intell.2016.07.001

**Published:** 2016

**Authors:** Elise Whitley, Ian J. Deary, Stuart J. Ritchie, G. David Batty, Meena Kumari, Michaela Benzeval

**Affiliations:** a Institute for Social and Economic Research, University of Essex, Colchester CO4 3SQ, UK; b MRC/CSO Social and Public Health Sciences Unit, University of Glasgow, Glasgow G12 8RZ, UK; c Centre for Cognitive Ageing and Cognitive Epidemiology, Department of Psychology, University of Edinburgh, Edinburgh, UK; d Department of Epidemiology and Public Health, University College London, London, UK

**Keywords:** Age differences, Aging, Cognitive ability, Memory

## Abstract

**Background:**

Populations worldwide are aging. Cognitive decline is an important precursor of dementia, illness and death and, even within the normal range, is associated with poorer performance on everyday tasks. However, the impact of age on cognitive function does not always receive the attention it deserves.

**Methods:**

We have explored cross-sectional associations of age with five cognitive tests (word recall, verbal fluency, subtraction, number sequence, and numerical problem solving) in a large representative sample of over 40,000 men and women aged 16 to 100 living in the UK.

**Results:**

Women performed better on word recall tests and men had higher scores for subtraction, number sequence and numerical problem solving. However, age-cognition associations were generally similar in both genders. Mean word recall and number sequence scores decreased from early adulthood with steeper declines from the mid-60s onwards Verbal fluency, subtraction and numerical problem solving scores remained stable or increased from early to mid-adulthood, followed by approximately linear declines from around age 60. Performance on all tests was progressively lower in respondents with increasingly worse self-rated health and memory. Age-related declines in word recall, verbal fluency and number sequence started earlier in those with the worst self-rated health. There was no compelling evidence for age dedifferentiation (that the general factor of cognitive ability changes in strength with age).

**Conclusions:**

We have confirmed previously observed patterns of cognitive aging using a large representative population sample.

## Highlights

• Cognitive performance by age varies by dimension but all are in decline by age 60.
• Gender differences in cognitive ability are generally consistent across all ages.
• Cognitive performance is lower in those with worse self-rated health and memory.
• Cognitive decline starts earlier in those with fair or poor self-rated health.
• There is very little evidence for age dedifferentiation of cognitive abilities.

## 1 Introduction

Improvements in living conditions, nutrition, social care, and medical technologies have led to a doubling in life expectancy in the last century (Ortman, Velkoff, & Hogan, 2014). For example, from 2012 to 2050, the United States is anticipated to experience a 53% rise in the population aged over 65 years and more than a doubling in those aged over 85 (Ortman et al., 2014). A similar transition is predicted in the United Kingdom (UK) and worldwide (Cracknell, 2010), and these demographic shifts have important implications for health, social care, and economic policy (Bloom et al., 2015, Commission of the European Communities, 2009, United Nations, 2002). Dementia is the most feared diagnoses for people aged over 50, with a recent survey in the UK reporting that 61% of respondents indicated that dementia was the condition they were most worried about, compared with 10% of people who put cancer first (Alzheimer's Society, 2012). In addition, a diagnosis of dementia or mild cognitive impairment has been shown to lead to anxiety and intense feelings of loss for patients and their families (Bunn et al., 2012). Cognitive decline is a common precursor of dementia, illness, institutionalisation, and death (Deary et al., 2009), and, even within the normal range, is associated with poorer performance on everyday tasks such as managing medication and finances (Tucker-Drob, 2011). Normative (i.e. non-pathological) age-related differences in cognition are therefore of interest in their own right and also in terms of the insights they offer into changes in brain and neurological function. In particular, it is important to understand the natural process of cognitive aging in order to identify how and when therapeutic interventions might best be applied, with large exercises such as the Dementias Platform UK recognising that the earliest stages of cognitive decline are the best ones to target in terms of prevention. However, in spite of its importance, the impact of age on cognitive function, as opposed to dementia or specific cognitive decline syndromes, does not always received the attention it deserves (Brayne, 2007, Hendrie et al., 2006).

Interventions aimed at preserving or improving cognitive function are often targeted at adults aged 60 or older (Williams & Kemper, 2010). However, changes in cognitive ability are not restricted to old age and are rather observed throughout the adult life-course (Foresight Mental Capital and Wellbeing Project, 2008). It is also the case that different domains of cognitive function peak and decline at different ages (Hartshorne and Germine, 2015, Salthouse, 2004, Salthouse, 2009a, Salthouse, 2009b, Salthouse, 2010, Schaie, 1994, Singh-Manoux et al., 2012). For example, while exact terminology varies, there is fairly consistent evidence of a steady decline from early to late adulthood in measures of fluid intelligence, such as efficiency or effectiveness of processing. In contrast, crystallised measures, for example vocabulary or information acquisition, tend to remain steady or increase until around age 60 years of age before declining thereafter. Notably, these associations of cognitive function with age are not necessarily linear and a comprehensive exploration of different dimensions of cognitive aging therefore requires data on a wide range of ages. Age-specific average scores for different cognitive dimensions have been presented previously in representative population samples of several thousand adults, e.g. those used to establish norms for the WAIS-IV, WMS-IV, and WJ-IV test batteries (Schrank et al., 2014, Wechsler, 2008, Wechsler, 2009). Results are also available from larger studies (Hartshorne and Germine, 2015, Salthouse, 2004, Salthouse, 2009a, Salthouse, 2009b, Schaie, 1994, Singh-Manoux et al., 2012), although these have not always been demonstrably representative.

Gender differences in cognitive abilities by age have been presented previously but results are mixed, variously suggesting greater age-related declines in women (Karlamangla et al., 2009, Meinz and Salthouse, 1998, Schaie, 1994, Van Dijk et al., 2008, Wu et al., 2012), greater declines in men (Capitani et al., 1998, Salthouse, 2014, Schaie, 1994, Zelinski and Gilewski, 2003), or consistent patterns in both genders (Ferreira, Ferreira Santos-Galduróz, Ferri, & Fernandes Galduróz, 2014).

Health has also been shown to impact on cognitive function, with several specific conditions and disease clusters reported to be associated with poorer cognitive function and cognitive decline (Aarts et al., 2011, van Boxtel et al., 1998). Self-rated health is a widely used health indicator that predicts morbidity and mortality (Jylha, 2009) while also allowing individuals to consider their health in the context of their own beliefs, priorities, experiences and circumstances. Previous reports of associations between self-rated health and cognitive function have, again, been mixed, with some suggesting worse cognitive function or faster cognitive decline in those with worse self-rated health (Carmelli et al., 1997, Sargent-Cox et al., 2011, Van Dijk et al., 2008), and others finding no association (Salthouse, 2014, Small et al., 2011). One aspect of self-rated health of particular relevance in this context is self-rated memory and, while there have been reports of negative associations with cognitive function (Amariglio et al., 2011, Begum et al., 2014, Genziani et al., 2013, Kim et al., 2003, Reid and Maclullich, 2006, Sargent-Cox et al., 2011, Wang et al., 2000), these have not always been consistent. Existing studies of self-rated health and memory are almost exclusively restricted to older adults, but there is no reason to suppose that similar effects will not be apparent at younger ages. In addition, existing results are generally based on a dichotomy of good versus poor self-rated health or memory and it is of considerable interest to establish whether differences in cognitive performance at different ages follow a gradient of worsening health or memory. However, analyses of this type require extremely large samples covering a wide range of ages.

Individuals who perform well on one cognitive test are also likely to perform well on others, which gives rise to a so-called positive manifold of correlations among mental tests. One long-standing and influential suggestion is that this arises because people differ on “general intelligence”, usually designated “*g*” and that this is reflected in different cognitive tests to different degrees (Spearman, 1904). It has been suggested that the *g*-saturation of manifest indicators (cognitive tests) may vary with age (Deary et al., 2010, Johnson et al., 2010, Salthouse, 2004) with, for example, vocabulary skills contributing to a greater extent and memory and processing speed to a lesser extent in older individuals. In addition, a ‘dedifferentiation’ hypothesis has been proposed, which posits that the proportion of variance in cognitive measures accounted for by *g* may increase with advancing age in adulthood (Deary et al., 1996, Tucker-Drob, 2009). Theoretically, it is posited that after becoming more specialized in early lifespan development (i.e. age differentiation), cognitive abilities may become more general (that is, their loadings on *g* will increase) in older age (age dedifferentiation), potentially due to generalised aging processes that affect or constrain different cognitive functions in similar ways (Tucker-Drob & Salthouse, 2008). The evidence supporting the cognitive dedifferentiation hypothesis (de Frias et al., 2007, Li et al., 2004) is mixed; some studies have failed to identify such an association (Anstey et al., 2003, Deary et al., 1996, Tucker-Drob and Salthouse, 2008, Tucker-Drob, 2009). Interestingly, one recent study found evidence for dedifferentiation of brain white matter tracts, which are known to underlie a portion of the variance in cognitive abilities (Cox et al., 2016; although in that study cognitive functions were not analysed). If important brain tissues are affected by the kinds of general aging processes mentioned above and therefore become more similar to each other, it may be that this has the effect of increasing the correlations among the cognitive abilities they support. However, as discussed previously, existing studies of cognitive dedifferentiation have tended to be limited by small sample sizes, unrepresentative populations, or restricted age ranges.

In the present analyses, we explore age variations in five dimensions of cognitive function in over 40,000 men and women aged 16 to 100 years living in the UK and sampled to be representative of the general population. In the context of examining cognition and age, this is an unusually large representative population-based sample. We aim to address five specific research questions. First, to what extent do age-related differences in different cognitive dimensions in our representative sample agree with those reported elsewhere? Second, given inconsistent results from previous samples, are age-related cognitive differences in these dimensions the same in men and in women? Third, again in the context of previous mixed results, does performance on different cognitive dimensions vary according to increasingly worse self-rated health at different ages, including in younger adults? Fourth, are previous reports of associations between poorer cognitive function in older adults with self-rated memory problems confirmed in different cognitive dimensions across a gradient of worsening self-rated memory, and are similar patterns observed in younger age groups? Finally, with regard to the age-associated dedifferentiation hypothesis, in the context of general intelligence, ‘*g*’, do the individual loadings and the portion of variance explained by *g* increase with age?

## 2 Methods

Analyses are based on data from *Understanding Society*, the UK Household Longitudinal Study (UKHLS) (Buck and McFall, 2011, University of Essex. Institute for Social and Economic Rese), details of which have been reported previously (Lynn, 2009). In brief, the UKHLS began in 2009 and is a longitudinal survey of 40,000 households in England, Scotland, Wales and Northern Ireland. Data are currently available from five collection waves. UKHLS combines four separate samples, one of which (the Innovation Panel, which carries methodological experiments) is not relevant here, and the current analyses are based on the other three. The largest of these samples is the General Population Sample (GPS) and is based on households drawn randomly from a stratified clustered sample of postcode sectors in England, Wales and Scotland and a random sample of postcode sectors in Northern Ireland, ensuring that they are representative of the UK population. The second sample is the Ethnic Minority Boost Sample (EMBS), which is an oversampling of the five main ethnic minority groups in the UK (Indian, Pakistani, Bangladeshi, Black Caribbean and Black African) so that at least 1000 adults from each group were included at wave 1. The third sample, which joined UKHLS in wave 2, is a sample of households from the British Household Panel Survey (BHPS), which has been running since 1991. Individuals from households in all samples are contacted annually to collect information on changes to their household and individual circumstances. If people join these households (flatmates, partners, parents etc.) they are included in the study for as long as they remain resident with an original sample member. Babies born to female original sample members also become permanent members of the study. If original household members leave and create new households they are included in the study. Sample members are followed and interviewed if they remain in the UK, and individuals are followed into institutional settings when practically possible.

GPS respondents recruited in wave 1 have been shown to be representative of the corresponding census population at the neighbourhood level (Petersen & Rabe, 2013). The impact of non-response and attrition in subsequent waves has been examined in detail (Lynn, Burton, Kaminska, Knies, & Nandi, 2012) and ongoing recruitment has been designed to maintain representativeness (Lynn, 2011). In addition, weights have been developed (Lynn & Kaminska, 2010) to ensure that analyses based on the three samples (GPS, EMB, BHPS) combined are also representative of the UK population. Results presented here are based on the three samples combined with analyses adjusted for sample design and attrition based cross-sectional inverse probability weights (Understanding Society, 2014) to ensure representativeness. Results based on the unweighted GPS sample, which is representative in its raw form (Petersen & Rabe, 2013) (not shown), were very similar.

### 2.1 Cognitive measures

In the third wave of data collection, carried out between January 2011 and April 2013, cognitive function measures were collected for respondents aged 16 and over (McFall, 2013) and these form the basis of the current analyses. Preliminary qualitative interviews were conducted with a group of 43 respondents to pilot the cognitive tests in different segments of the survey population (Gray, D'Ardenne, Balarajan, & Noah Uhrig, 2011). Following this, five measures were identified for inclusion in the main data collection, covering different domains of cognitive ability and skill. In the main survey, almost all (98.5%) interviews were face-to-face and, where this was not possible (1.5%), telephone interviews were carried out. A small number of respondents (1%) had cognitive tests translated into Arabic, Bengali, Cantonese, Gujarati, Punjabi in Gurmukhi or Urdu script, Somali, Urdu or Welsh. These were excluded from our analyses to avoid assumptions regarding comparability of cognitive tests in different languages.

Verbal declarative memory was assessed using both immediate and delayed word recall tasks. Respondents were asked to listen to a list of ten words delivered by a computer to ensure standardised delivery. They were then asked to recall the words immediately after the reading and, again, at a later stage in the interview without the words being repeated. The number of correct responses was recorded each time. Scores for the immediate and delayed recall tests (correlation coefficient: 0.76) were then summed to produce a single measure. This approach has been widely used elsewhere (e.g. the English Longitudinal Study of Ageing (ELSA) (Huppert, Gardener, & McWilliams, 2006), the US Health and Retirement Study (HRS) (Ofstedal, Fisher, & Herzog, 2005), the Survey of Health, Ageing and Retirement in Europe (SHARE) (Borsch-Supan et al., 2013), and the National Survey of Health and Development (NSHD) (Hurst et al., 2013)), and the word lists used here were those developed for the HRS.

Semantic verbal fluency was assessed by asking respondents to name as many animals as they could in one minute. The final score was based on the number of unique correct responses. This measure has also been used in ELSA (Llewellyn & Matthews, 2009), the German Socio-economic Panel Study (SOEP) (Lang, Weiss, Stocker, & von Rosenbladt, 2007), NSHD (Richards, Shipley, Fuhrer, & Wadsworth, 2004), and the Midlife in the United States study (MIDUS) (Lachman, Agrigoroaei, Murphy, & Tun, 2010).

In a subtraction test, again included in HRS (Ofstedal et al., 2005), and a component of screening instruments for cognitive impairment including the Mini Mental State Examination (Crum, Anthony, Bassett, & Folstein, 1993) and the Cambridge Cognitive Examination (CAMCOG) from the MRC Cognitive Function and Ageing study (CFAS) (Huppert, Brayne, Gill, Paykel, & Beardsall, 1995), respondents were asked to subtract 7 from 100 and then to subtract 7 from their answer on four more occasions. The number of correct responses out of a maximum of five was recorded.

Fluid reasoning was assessed using a measure again developed for the HRS (Fisher, McArdle, McCammon, Sonnega, & Weir, 2013), based on a small number of items from the Woodcock-Johnson tests of cognitive ability (Woodcock & Johnson, 1989), covering a range of difficulties. These items take the form of a number sequence in which the respondent is asked to fill in the gap(s) (e.g. 1, 2, _, 4). Respondents were initially presented with one or two simple examples to test their understanding; those who seemed confused or who did not appear to understand after two examples were not asked to complete the test. This approach resulted in a higher degree of missingness on this score compared with other measures. The implications of missingness are discussed below. Respondents who understood the problem moved onto the formal test, which involved two additional sets of three number sequences, with the difficulty of the second set determined by their performance on the first. Finally, a score was derived, based on the results from the two sets of tests, as described by HRS (Fisher et al., 2013). This score accounts for the difficulty level of the items and the probability that a respondent will answer correctly. In a normative sample, the mean score is 500 and an increase of 25% in the probability of getting an item right corresponds to a ten point increase in the score (McFall, 2013).

Numerical reasoning skills were based on a set of numerical problem solving questions taken from ELSA (Huppert et al., 2006) and also used by the HRS (Ofstedal et al., 2005) and SHARE (Banks, O'Dea, & Oldfield, 2010). Respondents were initially given three problems to solve and, depending on their responses, were then given a further one (simpler) or two (more difficult) problems. The total number of correct responses was recorded. Examples of problems presented to respondents include: calculating the sale price of a sofa in a half-price sale; working out change due from a simple monetary transaction; and, in the more difficult section, calculating compound interest over two years on a savings account earning 10% interest per year (McFall, 2013).

### 2.2 Other measures

Gender was recorded at the first wave of data collection. Age in years was recorded at the time of the cognitive testing in wave 3. Self-rated health was assessed in wave 3, using responses to “In general would you say your health is …” [excellent, very good, good, fair, or poor]. Self-rated health is a widely used health indicator and has been shown to predict morbidity and mortality (Jylha, 2009). Self-rated memory was based on the question “How would you rate your memory at the present time?” [excellent, very good, good, fair, or poor]. This question has been used in a number of other surveys, including ELSA (Huppert et al., 2006), HRS (Ofstedal et al., 2005), and the Irish Longitudinal Study on Ageing (TILDA) (Savva, Maty, Setti, & Feeney, 2013). As subjective measures, these questions may capture aspects of health and, in particular, cognitive function that are not covered by objective testing. In addition, these measures may reflect individual's personality type. For example, individuals with greater negative effect have been shown to give more pessimistic reports of their health (Benyamini et al., 2000, Blaxter, 1990, Blaxter, 2004, Kraus et al., 2013).

### 2.3 Statistical methods

All cognitive measures were transformed into standardised z-scores, which represent the number of standard deviations an individual's score lies above or below the population mean. Mean (95% confidence interval) z-scores were calculated in one-year age groups from 16 to 89; there were fewer respondents aged 90 years or above and they were combined into a single category. Results are presented separately for men and women and according to self-rated health and self-rated memory. We also used confirmatory factor analysis to test whether there were similar general factors of cognitive ability in men and women: using the method described by Widaman, Ferrer, and Conger (2010), we tested whether a multi-group model of *g* split by sex was better-fitting with configural, weak, or strong measurement invariance across the sexes. To do this, we used the chi-squared test as well as comparison of the models' Akaike Information Criteria (AIC) and Bayesian Information Criteria (BIC). These models were run using Mplus v7.3 (Muthén & Muthén, 1998–2014).

We also explored age-related changes in *g*, extracted from the scores on the five tests using confirmatory factor analysis, and considered how the *g*-factor scores differed according to gender and levels of self-rated health and memory. Preliminary analyses were carried out based on all five self-rated health and memory categories but relatively few respondents rated their health as poor or their memory as poor or excellent and results in these groups were therefore less robust, with considerably wider confidence intervals (CI), than those based on other categories. However, within these limitations, results for those rating their health or memory as excellent were very similar to those rating them as very good, and the same was true for respondents rating their health or memory as fair and poor. For ease of interpretation we therefore present results for self-rated health and memory in three categories (excellent or very good versus good versus fair or poor). Results for all five categories of self-rated health and memory are presented in the supplementary materials.

To address the age dedifferentiation hypothesis, we used two methods to test whether the general factor of cognitive ability (*g*) varied with age. First, we used locally weighted structural equation modelling (LOSEM) (Hildebrandt, Wilhelm, & Robitzch, 2009) to produce graphics in which to observe non-parametric age trends in factor loadings (the correlation of each test with *g*), uniquenesses (the residual variance in each test not accounted for by *g*), and communalities (the portion of the total variance in each test accounted for by *g*, i.e. the squared loading divided by the squared uniqueness plus the squared loading). This analysis was performed using the MplusAutomation package for R (Hallquist & Wiley, 2014), which allows batch running of models in Mplus. Second, we used a parametric method to test for de-differentiation described by Cox et al. (2016) and Cheung, Harden, and Tucker-Drob (2015) (see also Molenaar, Dolan, Wicherts, & van der Maas, 2010 for a somewhat different approach that focusses on total factor variances; Tucker-Drob, 2009, for an earlier implementation of the approach used here). This method tests dedifferentiation within an age-moderated factor analysis. In this model, *g* is estimated from the cognitive tests in a confirmatory factor analysis, and age is included as a moderator of each individual test's factor loading and each test-specific uniqueness. The model thus produces a parameter for the main effect of age and a parameter for the age moderation effect for the loading and uniqueness for each test. The model can be written, following Cox et al. (2016), as:

*Y*[*t*]_*n*_ = υ[*t*] + α_1_[*t*] × *age*_*n*_ + α_2_[*t*] × *sex*_*n*_ + (λ_1_[*t*] + λ_1_ ′ [*t*] × *age*_*n*_) × *g*_*n*_ + (λ_2_[*t*] + λ_2_ ′ [*t*] × *age*_*n*_) × *u*[*t*]_*n*_

where *Y*[*t*] represents an individual's score on cognitive test [*t*], of which there were five (*Y*[Word Recall], *Y*[Subtraction], *Y*[Number Sequence], *Y*[Verbal Fluency], and *Y*[Numerical Ability]); υ[*t*] is a test-specific regression intercept; α_1_[*t*] and α_2_[*t*] are test-specific regression coefficients for the effects of age and sex, respectively; λ_1_[t] is a test-specific loading (main effect) on the general factor of cognitive ability (*g*); λ_2_[*t*] is a test-specific loading (main effect) on the test-specific uniqueness factor (*u*[*t*]); λ_1_[*t*]′ is a test-specific interaction parameter representing age moderation of the loadings on the general factor, and λ_2_[*t*]′ is a test-specific interaction parameters representing age moderation of the test-specific uniqueness factor, respectively. Subscript _*n*_ indicates that a variable varies across individuals. Of interest here is whether the interaction parameters are significantly different from zero; that is, whether the tests' loadings and/or uniquenesses differ significantly with age. The communality for each cognitive test can be calculated from the loadings and uniquenesses as described above, and the mean communality across all tests indicates the proportion of variance across all the tests explained by *g*. These models were also run in Mplus v7.3.

### 2.4 Sensitivity analyses

Results presented here are based on respondents with complete data for all five cognitive measures to allow comparison between them. However, results based on those with complete data for individual cognitive measures were very similar. In addition, although scores from tests of word recall, verbal fluency and number sequence were all approximately normally distributed, there was some evidence of ceiling effects in tests of numerical ability and, particularly, subtraction for which over half of respondents calculated all five subtractions correctly. We therefore repeated our analyses of age-related changes in *g* excluding these two tests to explore the potential impact of these ceiling effects. Results from these analyses, although based on fewer tests, were similar to those presented here.

## 3 Results

In total, 49,258 respondents aged 16 years or older were interviewed in English in the third wave of data collection. Of these, 33,165 (67.3%) were from the GPS, 11,318 (23.0%) from the BHPS, and 4775 (9.7) from the EMBS (Table 1, unweighted). The gender breakdown of the three samples was almost identical, with around 54% of respondents female. The age distributions of the GPS and BHPS samples were also very similar, while respondents from the EMBS tended to be younger (mean age 39 years compared with 48 years in GPS/BHPS). Self-rated health was broadly similar across the three samples with just under a fifth rating their health as excellent, around a third very good, a quarter good, and around 15% fair and 6% poor. Self-rated memory was similar in the GPS and BHPS samples (4% excellent, around a fifth very good, just over a third good, around a quarter fair and approximately 10% poor). Respondents in the EMBS were generally more positive about their memory, possibly reflecting the age differences between the samples. Around a fifth to a quarter of all respondents had no qualifications; however BHPS respondents were less likely to have post-school qualifications (27%) compared with GPS (35%) and EMBS (41%) respondents.

Cognitive data were available for between 41,926 (85%; number sequence test) and 44,746 (91%; verbal fluency) respondents, depending on the test in question. Mean cognition scores were similar in the GPS and BHPS and somewhat lower in the EMBS (Table 1; e.g. mean (SD) word recall score in GPS, BHPS and EMBS: 11.5 (3.6), 11.4 (3.7) and 11.0 (3.6) respectively). A total of 40,730 (83%) respondents had complete data for all five cognitive measures. Compared with respondents with complete data, those with one or more missing cognitive measure were slightly older, and were more likely to be male, less educated, have poorer self-rated health and memory, and, where data were available, scored lower on other cognitive tests (Table 2, unweighted).

**Fig. 1:**
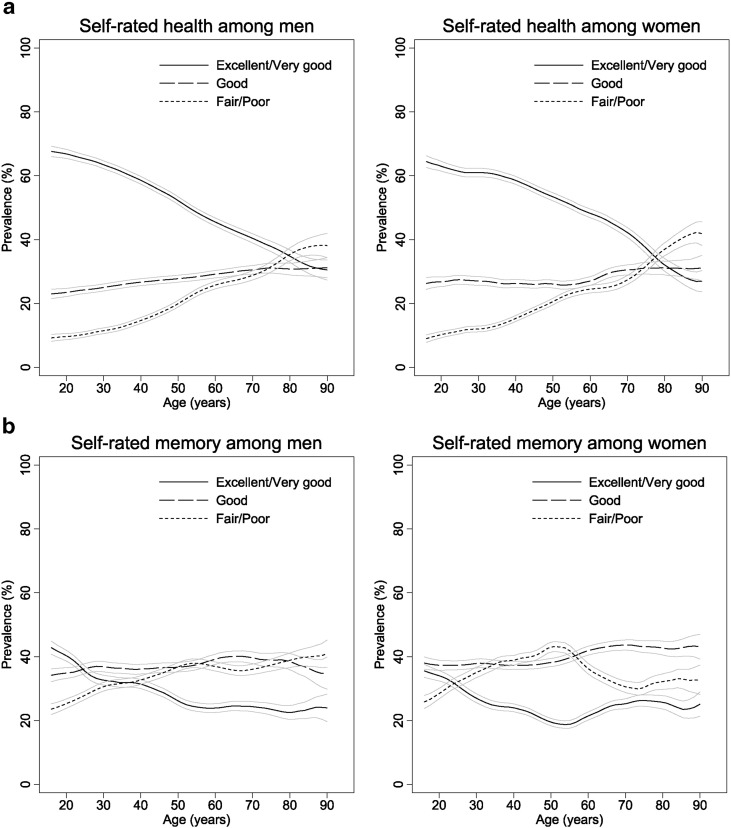
a: Prevalence of self-rated health by age among men and women b: Prevalence of self-rated memory by age among men and women.

Prevalences of self-rated health and memory are presented by age (in one-year groups) and gender Fig. 1 and b respectively. Self-rated health was broadly similar in both genders, with the prevalence of excellent or very good health decreasing steadily with increasing age ( reduction in prevalence (95% CI) per additional five years of age = 3.1% (2.9%, 3.2%) and 2.5% (2.3%, 2.6%) in men and women respectively). There were corresponding increases in the prevalence of good and, most markedly, fair or poor self-rated health with increasing age, (increase in prevalence of fair or poor self-rated health per additional five years of age = 2.3% (2.1%, 2.4%) and 2.2% (2.1%, 2.3%) in men and women respectively).

The proportion of men and women rating their memory as good remained fairly consistent across all ages. However, the age-specific prevalence of excellent/very good and fair/poor self-rated memory differed between men and women. The prevalence of excellent or very good memory in men decreased fairly steadily with increasing age (reduction in prevalence per additional five years of age = 1.5% (1.3%, 1.7%)) with corresponding increases in those rating their memory as fair or poor (increase in prevalence per additional five years of age = 1.2% (1.0%, 1.3%)). In women, similar patterns of decreasing excellent/very good and increasing fair/poor self-rated memory were observed up to around age 50 or 55 but were apparently reversed thereafter up to around age 70 before levelling out at later ages (difference in prevalence of excellent or very good self-rated memory per additional five years of age = − 2.3% (− 2.6%, − 2.0%) and 2.6% (1.4%, 3.9%) at ages < 50 and 55–70 respectively; difference in prevalence of fair or poor self-rated memory per additional five years of age = 2.3% (1.9%, 2.8%) and − 4.3% (− 5.2%, − 3.4%) at ages < 50 and 50–70 respectively).

**Fig. 2:**
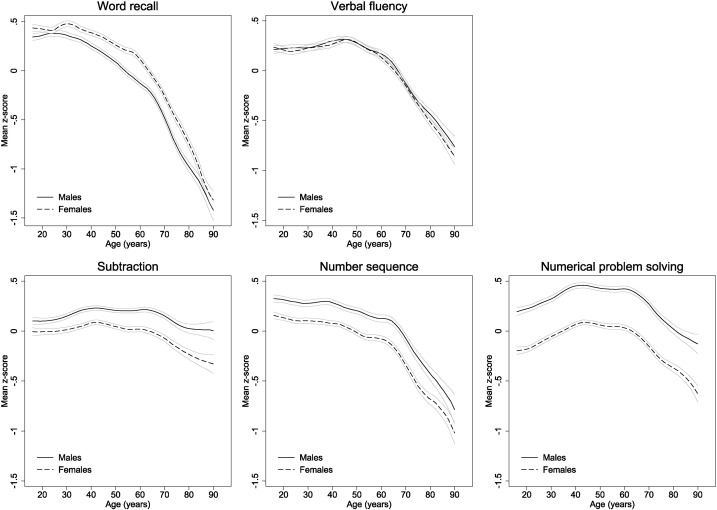
Mean standardised z-scores for all five cognitive measures by age and gender.

Mean cognitive measure z-scores by age (in one year groups) and gender are presented in Fig. 2. There were clear differences in the patterns of cognitive function by age according to the different cognitive measures. Word recall, was generally higher in women than men (difference in z-score in women versus men = 0.15 (0.14, 0.17)) and declined in both males and females from around age 30 years, steadily at first (reduction in z-score per additional five years of age = 0.07 (0.07, 0.08)) and then more rapidly from around age 60 (reduction in z-score per additional five years of age = 0.24 (0.23, 0.25)). The initial decline in word recall was more marked in males although, by the mid-80s, z-scores were similar in both genders. Verbal fluency was very similar in both genders at all ages and remained fairly consistent up to around age 50 before declining with increasing age, particularly from the mid-60s onwards (reduction in z-score per additional five years of age = 0.17 (0.16, 0.18)). There were marked gender differences in the three remaining dimensions with z-scores for subtraction, number sequence, and numerical problem solving in men 0.18 (0.16, 0.19), 0.21 (0.19, 0.23), and 0.39 (0.37, 0.41) higher, respectively, on average. However, differences by age generally followed very similar patterns in both genders. Subtraction scores increased slightly at early ages and then remained constant up to the mid-60s before declining steadily thereafter (decrease in z-score per additional five years = 0.06 (0.04, 0.08)). Scores for number sequence declined slightly but steadily up to the mid-60s (decrease per additional five years of age = 0.02 (0.02, 0.03)) and showed a marked decline thereafter (0.18 (0.16, 0.20)). Numerical problem solving ability increased steadily in both genders up to around age 40 (increase per additional five years of age = 0.06 (0.05, 0.07)), remained fairly constant up to age 60, and declined thereafter (decrease in z-score per five additional years of age = 0.11 (0.10, 0.12)).

We next tested measurement invariance by sex. A multi-group confirmatory factor analysis showed that a model with configural invariance across the sexes had excellent fit to the data (*χ*^2^(8) = 283.81, *p* < 0.001, RMSEA = 0.041, CFI = 0.993, TLI = 0.981), as did a model with weak invariance (*χ*^2^(12) = 476.23, *p* < 0.001, RMSEA = 0.044, CFI = 0.988, TLI = 0.979). However, the model with strong invariance had poorer fit (*χ*^2^(17) = 3668.12, *p* < 0.001, RMSEA = 0.103, CFI = 0.902, TLI = 0.884); indeed, the model with only configural invariance had significantly better fit than either the weak (*χ*^2^(4) = 192.42, *p* < 0.001, ΔAIC = 184.42, ΔBIC = 149.96) or strong (*χ*^2^(11) = 3384.32, *p* < 0.001, ΔAIC = 3366.32, ΔBIC = 3288.78) invariance models. Thus, there was evidence that the *g*-factor of cognitive ability had different structure across the sexes.

**Fig. 3:**
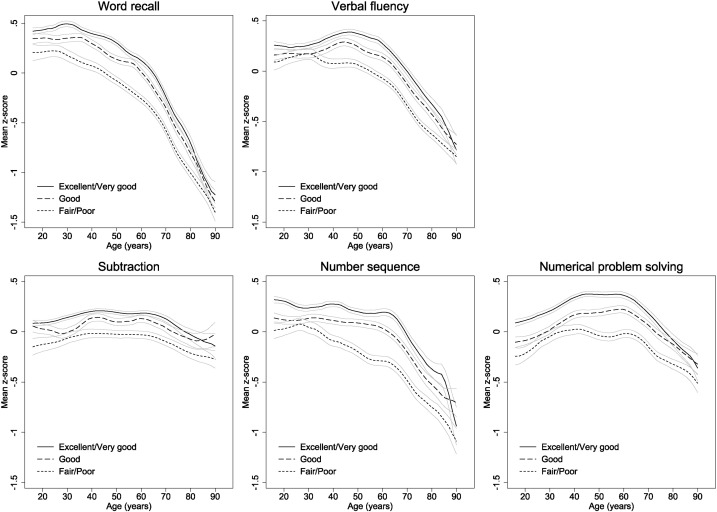
Mean standardised z-scores for all five cognitive measures by age and self-rated health.

Fig. 3 presents age-specific mean cognitive z-scores separately for respondents according to self-rated health. Individuals with increasingly poorer self-rated health performed progressively worse on all cognitive measures at all ages up to around 85, with mean z-scores in those with excellent or very good self-rated health between 0.23 (0.21, 0.26) (subtraction) and 0.39 (0.37, 0.42) (number sequence) higher on average than in those with fair or poor self-rated health. These differences were generally greatest in middle- to early old-age. Decreases in word recall, verbal fluency, number sequence, and numerical ability appeared to start earlier in those with poor or fair self-rated health (at age 25 versus 30 or 35 for word recall, age 30 versus 45 for verbal fluency, age 30 versus 45 for number sequence, and age 40 versus 60 for numerical ability) and, for some cognitive measures, these early decreases were mirrored by increases in z-scores in those with good, very good, or excellent self-rated health at the same ages (e.g. difference in verbal fluency z-score per additional five years of age between 30 and 45 = 0.04 (0.01, 0.07) and − 0.07 (− 0.12, − 0.02) in those with very good or excellent versus poor or fair self-rated health respectively). By age 85 differences were substantially reduced and, in some cases, no longer apparent. Gender-specific trends with age in those with good or better self-rated health were very similar to those presented in Fig. 1 (see Supplementary materials).

**Fig. 4:**
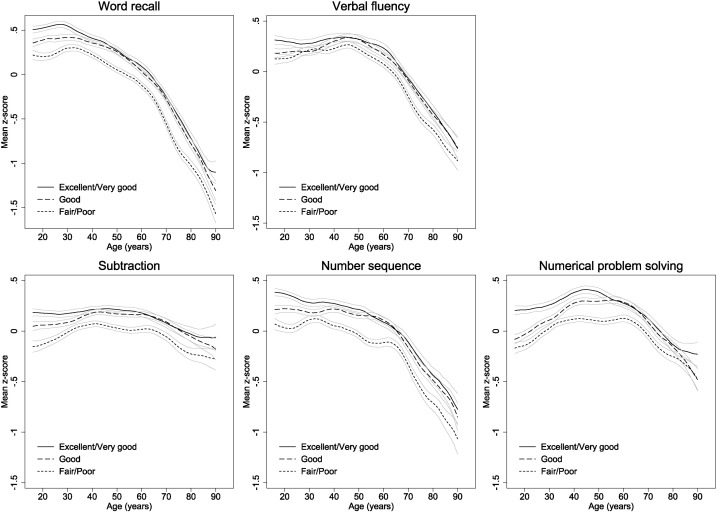
Mean standardised z-scores for all five cognitive measures by age and self-rated memory.

Mean cognitive z-scores by age are presented separately according to self-rated memory in Fig. 4. Differences were less marked than those for self-rated health but, again, mean z-scores were lowest in those with fair or poor self-rated memory, with differences between those with fair or poor versus very good or excellent self-rated memory of between 0.10 (0.07, 0.12) (verbal fluency) and 0.22 (0.19, 0.24) (number sequence). Younger respondents with good self-rated memory had lower cognitive z-scores than those with very good or excellent self-rated memory but these differences did not continue past ages 35–40 or, in the case of numerical ability, age 50. Differences between those with fair or poor versus better self-rated memory were broadly consistent at all ages and patterns of cognitive function with age were similar in those with fair/poor, good and very good/excellent self-rated memory. The exception to this was numerical problem solving for which increases in z-scores at early ages stopped earlier in those with fair or poor self-rated memory (at around 35 versus 45 in those with good or better self-rated memory), and the decrease in z-scores at older ages started much earlier in those rating their memory as very good or excellent (at around age 45 versus 60 in those with good, fair or poor self-rated memory), although z-scores in those with very good or excellent self-rated memory were higher at all ages. Gender-specific patterns with age in those with good or better self-rated memory were very similar to those for all respondents combined (see Supplementary materials).

**Fig. 5:**
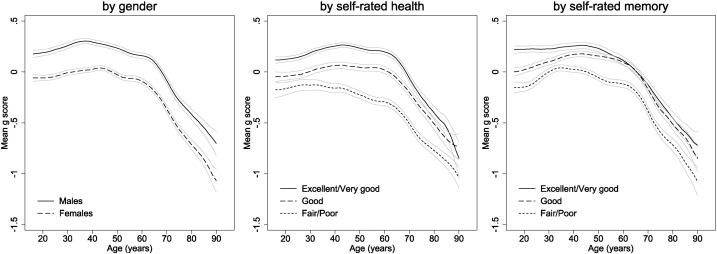
Mean g scores by age and gender, self-rated health, and self-rated memory.

Confirmatory factor analyses were then performed to investigate the tests' relations with *g*. Across the full sample, the standardised *g*-loadings (standard error (SE)) for each test in a one-factor model were as follows: word recall = 0.45 (0.005); verbal fluency = 0.45 (0.005); subtraction = 0.52 (0.005); number sequence = 0.72 (0.004); numerical problem solving = 0.71 (0.004). We included one correlated residual in the model to account for content overlap between word recall and verbal fluency (standardised *β* = 0.24, SE = 0.005). The model had excellent fit to the data: *χ*^2^(4) = 282.94, Root Mean Square Error of Approximation = 0.04, Comparative Fit Index = 0.99, Tucker-Lewis Index = 0.98. Mean *g* scores by age are presented according to gender and self-rated health and memory in Fig. 5. Scores were consistently lower in women with early rises in both genders followed by slow declines (from around ages 35 and 45 in men and women respectively) and more rapid declines from early to mid-60s in both genders. Individuals with poorer self-rated health or memory had progressively lower g scores, with declines in scores starting 20 or 30 years earlier in those with fair or poor versus good or better self-rated health or memory. This one-factor model was used as the basis for the two differentiation analyses described below.

**Fig. 6:**
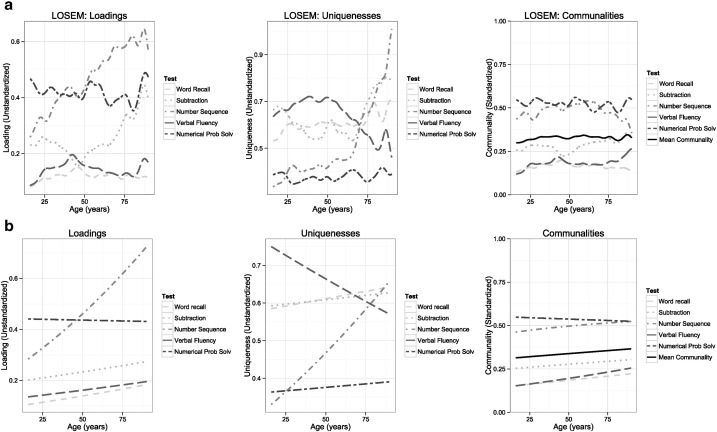
*g*-Factor loadings (left), uniquenesses (middle), and communalities (right) estimated using (a) LOSEM and (b) moderated factor analysis for the five cognitive tests.

The results of the dedifferentiation analyses are illustrated in Fig. 6. Results using LOSEM are presented in the top row. Note that this analysis used as its basis the factor model of g with the fit statistics described in the previous paragraph. Whereas some cognitive measures (specifically number sequence and subtraction) showed substantially higher *g*-loadings in older participants, for these variables the uniquenesses also showed an increase. That is, the overall amount of variance increased and, as a result, the communalities were generally flat across the age span. As can be seen from the solid line in the rightmost plot, the mean communality showed little substantial change with age (indicating that the overall variance across the cognitive tests accounted for by *g* was fairly similar at all ages). The second, parametric differentiation analysis produced highly comparable results to the LOSEM, as shown in the bottom row (see supplementary materials for full results with statistical significance testing for loadings and communalities). The mean communality showed a slight increase, beginning at 0.31 at age 16 years and increasing to 0.37 at age 90 years (that is, 5.2% more of the variance across the tests was explained by *g* at age 90 than age 16). These very low estimates provided little evidence for age dedifferentiation in our analyses.

## 4 Discussion

We have explored five cognitive measures in one-year age groups in a large representative population-based sample of over 40,000 UK men and women aged 16 to 100. We observed earlier decreases in cognitive function for measures of processing efficiency or effectiveness and later decreases or early increases in knowledge-based measures followed by steeper, approximately linear declines in all measures from around age 60. These results confirm those reported previously (Hartshorne and Germine, 2015, Salthouse, 2004, Salthouse, 2009b, Salthouse, 2010, Schaie, 1994, Singh-Manoux et al., 2012). We found little evidence for dedifferentiation, i.e. that the percentage of variance in cognitive measures accounted for by *g* increases with advancing age.

Existing studies that present age-specific results separately by gender have often been restricted to overall cognitive performance or a single cognitive measure, making it difficult to compare age-related changes over different cognitive dimensions. In addition, those based on representative samples have often focussed on older adults. However, there is some existing evidence to suggest that women may perform better on tests based on verbal meaning while men may have more ability in numerical tests (Schaie, 1994). We observed markedly higher scores in numerical tests in men, which would be consistent with previous evidence, but there was no obvious gender difference in verbal fluency. It has been hypothesised that apparent male advantages in cognitive ability may be a result of common biases in selection effects, with men and individuals of lower intelligence tending to be under-represented in most population surveys (Dykiert et al., 2009, Madhyastha et al., 2009). Our analytical sample (restricted to those with complete data for all cognitive measures) followed this pattern and it is therefore plausible that the observed gender differences in numerical tests and lack of gender difference in verbal fluency may have been driven, at least in part, by under-sampling men with lower cognitive ability. What is striking about our results is that differences by age were highly similar in both genders, particularly at ages < 80 years, with the possible exception of a more marked decline in word recall in men. Our sex difference results in terms of the *g*-factor must be seen in the light of our analysis of measurement invariance: there was no measurement invariance across the sexes, meaning that any true sex differences in general cognitive ability are difficult to interpret. Although small differences in model fit were exaggerated due to our large sample size, models with both weak and strong sex invariance had significantly poorer fit, indicating that the makeup of the *g*-factor was not the same by sex.

Our analyses by self-rated health and memory expand on previous work by considering the full range of possible responses rather than the more commonly presented dichotomy of “good” versus “poor” health or memory. Our results suggest that cognitive ability was increasingly lower in those who reported progressively poorer health at all ages, although differences according to self-rated health were reduced from around age 85 onwards, possibly reflecting the increasing impact of differential mortality due to cognitive ability at these older ages. Consistent with previous evidence in older age groups (Carmelli et al., 1997, Sargent-Cox et al., 2011, Van Dijk et al., 2008), we also observed earlier age-related declines in some cognitive dimensions and our results extend these previous findings by also considering younger ages. Specifically our results suggest that declines in word recall, verbal fluency and number sequence started between 10 and 15 years earlier in those reporting poor or fair versus good or better health, with declines starting around or even before age 30. This is an interesting result, although the use of cross-sectional data in this context means that we can't rule out greater reporting of poorer health in those with lower cognitive function. Results restricted to those with good self-rated health were very similar to those based on all respondents, suggesting that the observed declines in cognitive functioning in all respondents combined were not simply due to worsening health in older individuals.

A recent review of empirical evidence concluded that self-rated memory correlates positively, although weakly, with objective measures of memory and that the extent of agreement varies by age, gender, education and depression (Crumley, Stetler, & Horhota, 2014). Declining memory is popularly regarded as an inevitable consequence of aging and this may lead to a mismatch between perceived and actual memory performance in older adults. Specifically, while many older adults consider their memory to be poorer than those younger than themselves, the majority see no difference between their own (potentially declining) memory and that of their peers (Lineweaver & Hertzog, 1998). Our results are consistent with this phenomenon, with self-rated memory declining at a markedly slower pace than objective measures of memory and, in the case of women, levelling off or even potentially improving at later ages, indicating that the experiences and expectations underlying individual's assessment of their own memory may also change with age. However, it is of note that performance on cognitive tests was consistently lower in respondents with poor self-rated memory, with some of the greatest differences apparent in younger respondents, who have not been widely considered in the past. This suggests that, while individuals may be broadly optimistic about their memory in the context of their age, they can also be realistic about limitations to their cognitive functioning.

Previous evidence suggests that the contribution of different cognitive dimensions to general intelligence, *g*, may vary by age (Deary et al., 2010, Johnson et al., 2010, Salthouse, 2004), while the dedifferentiation hypothesis states that the percentage of variance in cognitive measures accounted for by *g* may increase with advancing age in adulthood (Deary et al., 1996, Tucker-Drob, 2009). We found very little support for either of these ideas in our sample: test communalities remained generally stable with age, showing only very small increases. These results suggest that the *g*-factor of cognitive ability retains a similar strength across most of the adult lifespan.

### 4.1 Strengths and limitations

Our analyses are based on data collected from a substantial population-based sample of over 40,000 respondents and cognitive tests were selected, in part, for brevity. This means that participants did not complete a full cognitive battery and some cognitive abilities that require longer testing sessions (such as abstract reasoning or comprehension as measured by Wechsler-type tests) were not assessed. This may have impacted on our calculation of the *g*-factor and, although five tests is sufficient to allow an adequate common factor, additional tests would have allowed a more accurate and generalisable investigation of *g*. Ceiling effects in tests of numerical ability and, particularly, subtraction mean that we were unable to distinguish well between respondents at the upper end of these two dimensions. This may have somewhat limited our ability to detect age-related changes and may explain the apparently weaker results for subtraction and numerical ability. However, these tests have been used widely in other surveys, as described above, have been shown to be reliable and valid (McFall, 2013), and analyses of *g* excluding these dimensions were similar to those presented here. Ceiling effects were not apparent in the other three dimensions.

Our results are based on cross-sectional data, which offers substantially greater numbers of individuals across all ages from 16 to 90 + than would be easily available using a longitudinal approach. Previous analyses of age-related changes in cognition based on cross-sectional data generally indicate an earlier and steeper decline than those based on longitudinal data and the debate regarding the possible explanations for this discrepancy and the relative merits of the two approaches has been well rehearsed in the literature (Rönnlund et al., 2005, Salthouse, 2009b). The principal disadvantage of cross-sectional data in this context is that results may be affected by cohort effects, while those based on longitudinal data may be influenced by retest or practise effects. Recent work has focussed on quantifying the impact of retest biases, and longitudinal analyses adjusted for retest effects are generally closer to those based on cross-sectional, non-human and neurobiological data (Salthouse, 2009b, Salthouse, 2013a, Salthouse, 2013b). Although we cannot rule out some impact of cohort effects it is worth noting that our results are consistent with those reported elsewhere for specific age ranges and populations, and also that the differences in cognition that we observed tended to be evident year-on-year while any cohort effects would be likely to take place over longer periods. Future plans to repeat cognitive testing in subsequent waves of data collection will allow us to explore this phenomenon in more detail.

The greatest strength of the present analyses is the representativeness of the samples combined with size. Both weighted analyses of UKHLS and those (unweighted) based on the GPS subsample have been shown to be representative of the UK population (Lynn, 2011, Lynn et al., 2012, Petersen and Rabe, 2013). The results presented here are based on those respondents with complete data for all cognitive tests; those with missing cognitive scores were slightly older, had fewer educational qualifications, and tended to perform worse on other cognitive tests (where these results were available). In addition, greater mortality during follow-up in individuals with lower socioeconomic position may also have resulted in fewer less educated individuals at older ages although, as cognitive data were collected only three years from baseline, such effects are likely to be small. The over-representation of younger, higher ability individuals in studies of cognitive aging is well established. However, our original samples were fully representative of the UK population and we therefore believe that the impact of these losses will be limited. It is also worth noting that analyses of individual cognitive measures, which involved fewer missing data, were very similar to those presented here. These limitations are likely to have resulted in an under-representation of individuals with lower cognitive scores at older ages, meaning that, if anything, we may have underestimated the true rate of cognitive decline with age.

### 4.2 Conclusion

We have described age-related differences in five different cognitive measures in a substantial representative population sample of over 40,000 individuals aged 16 to 90 +. We found that scores for word recall and number sequence declined steadily from early adulthood, while scores for verbal fluency, subtraction and numerical problem solving only decreased after around age 60. In addition, we observed increasingly poorer cognitive performance in respondents with progressively worse self-rated health and memory, with cognitive declines in the worst rated groups beginning in early adulthood.

## Figures and Tables

**Table 1:** Wave 3 characteristics of Understanding Society (UKHLS) participants by sub-sample.

	General Population Sample (GPS)(N⁎ = 33,165)	British Household Panel Survey (BHPS) (N⁎ = 11,318)	Ethnic Minority Boost (EMB) (N⁎ = 4,775)
Gender (n (%))			
Male	15,201 (45.8)	5,178 (45.8)	2,199 (46.1)
Female	17,964 (54.2)	6,140 (54.3)	2,576 (54.0)
Age in years (mean (SD))	48.1 (18.5)	47.8 (19.0)	38.8 (15.6)
Self-rated health (n (%))⁎⁎			
Excellent	5,875 (17.7)	1,932 (17.1)	901 (18.9)
Very good	11,445 (34.5)	3,834 (33.9)	1,537 (32.3)
Good	8,773 (26.5)	3,048 (27.0)	1,349 (28.3)
Fair	4,893 (14.8)	1,778 (15.7)	671 (14.1)
Poor	2,155 (6.5)	718 (6.4)	306 (6.4)
Self-rated memory (n (%))⁎⁎			
Excellent	1,332 (4.4)	423 (4.0)	305 (7.5)
Very good	6,672 (21.9)	2,210 (20.7)	1,032 (25.3)
Good	11,489 (37.7)	4,015 (37.6)	1,540 (37.7)
Fair	8,167 (26.8)	2,993 (28.0)	871 (21.3)
Poor	2,842 (9.3)	1,047 (9.8)	335 (8.2)
Highest qualification (n (%))⁎⁎			
No qualifications	8,053 (24.6)	2,684 (25.0)	918 (19.5)
School level	13,326 (40.7)	5,162 (48.1)	1,879 (39.9)
Degree/professional or higher	11,353 (34.7)	2,898 (27.0)	1,918 (40.7)
Cognitive measures (mean (SD))			
Word recall (range 0–20)	11.5 (3.6)	11.4 (3.7)	11.0 (3.6)
Verbal fluency (range 0–99)	21.8 (7.0)	21.5 (7.1)	17.9 (6.8)
Subtraction (range 0–5)	4.4 (1.1)	4.5 (1.0)	4.1 (1.3)
Number sequence (range 409–584)	529.8 (31.7)	530.7 (31.5)	521.2 (36.0)
Numerical problem solving (range 0–5)	3.6 (1.1)	3.6 (1.1)	3.1 (1.2)

⁎ Raw numbers, i.e. unweighted.

⁎⁎ Numbers do not add up to totals in column titles due to missing values.

**Table 2:** Comparison of UKHLS participants with complete versus missing cognitive data.

	Participants with complete cognitive data (N⁎ = 40,730)	Participants with one or more missing cognitive measure (N⁎ = 8,528)
Gender (n (%))		
Male	18,160 (44.6)	4,418 (51.8)
Female	22,570 (55.4)	4,110 (48.2)
Age (mean (SD))	46.9 (18.1)	48.0 (20.6)
Self-rated health (n (%))⁎⁎		
Excellent	7,185 (17.6)	1,523 (17.9)
Very good	14,474 (35.5)	2,342 (27.6)
Good	11,126 (27.3)	2,044 (24.1)
Fair	5,817 (14.3)	1,525 (18.0)
Poor	2120 (5.2)	1,059 (12.5)
Self-rated memory (n (%))⁎⁎		
Excellent	1,867 (4.6)	193 (4.2)
Very good	9,188 (22.6)	726 (16.0)
Good	15,564 (38.2)	1,480 (32.6)
Fair	10,709 (26.3)	1,322 (29.1)
Poor	3,401 (8.4)	823 (18.1)
Highest qualification (n (%))⁎⁎		
No qualifications	8,597 (21.4)	3,058 (38.3)
School level	17,280 (43.0)	3,087 (38.6)
Degree/professional or higher	14,325 (35.6)	1,844 (23.1)
Cognitive test z-score (mean (SD))		
Word recall	0.09 (0.93)	− 1.06 (1.21)
Verbal fluency	0.10 (0.93)	− 0.89 (1.19)
Subtraction	0.07 (0.91)	− 0.84 (1.52)
Number sequence	0.03 (0.97)	− 0.73 (1.38)
Numerical problem solving	0.11 (0.90)	− 1.10 (1.25)

⁎ Raw numbers, i.e. unweighted.

⁎⁎ Numbers do not add up to total due to missing values of descriptive variables.

## Appendix A Supplementary data

Supplementary analyses. Image 1
