# Supplementary material for: Variations in cognitive abilities across the life course: Cross-sectional evidence from Understanding Society: The UK Household Longitudinal Study
Source: Intelligence. 2016 Nov-Dec;59:39–50. doi: 10.1016/j.intell.2016.07.001 (PMC5127898; doi:10.1016/j.intell.2016.07.001)
Supplement: Supplementary file 1 — Supplementary analyses. [file mmc1.docx]

Supplementary materials Figure S1: Prevalence of self-rated health in five categories by age among men and women

Supplementary materials Figure S2: Prevalence of self-rated memory in five categories by age among men and women

Supplementary materials Figure S3: Mean standardised z-scores for all five cognitive measures by age and self-rated health

Supplementary materials Figure S4: Mean standardised z-scores for all five cognitive measures by age and self-rated memory

Supplementary materials Figure S5: Mean standardised z-scores for all five cognitive measures by age and gender *in those with good or better self-rated health*

Supplementary materials Figure S6: Mean standardised z-scores for all five cognitive measures by age and gender *in those with good or better self-rated memory*

Supplementary materials Table S1: Parameter estimates and statistical significance tests for estimates of the main effects of age (λ_1_ and λ_2_ in Equation 1) and the age moderation (λ_1_′ and λ_2_′ in Equation 1) parameters for *g*-factor loadings and test uniquenesses in the moderated factor analysis model. All estimates are unstandardized.

| Test | Age parameter | Factor loading | | | Uniqueness | | |
| --- | --- | --- | --- | --- | --- | --- | --- |
|  |  | Estimate | SE | *p*-value | Estimate | SE | *p*-value |
| Word recall | Main effect | 0.362 | 0.009 | <0.001 | 0.765 | 0.006 | <0.001 |
|  | Moderation | 0.014 | 0.003 | <0.001 | 0.005 | 0.002 | 0.001 |
| Verbal fluency | Main effect | 0.368 | 0.010 | <0.001 | 0.886 | 0.006 | <0.001 |
|  | Moderation | 0.010 | 0.003 | <0.001 | -0.015 | 0.002 | <0.001 |
| Subtraction | Main effect | 0.449 | 0.010 | <0.001 | 0.770 | 0.006 | <0.001 |
|  | Moderation | 0.010 | 0.003 | <0.001 | 0.003 | 0.002 | 0.100 |
| Number sequence | Main effect | 0.553 | 0.009 | <0.001 | 0.575 | 0.007 | <0.001 |
|  | Moderation | 0.043 | 0.003 | <0.001 | 0.032 | 0.002 | <0.001 |
| Numerical problem solving | Main effect | 0.664 | 0.010 | <0.001 | 0.603 | 0.008 | <0.001 |
|  | Moderation | -0.001 | 0.003 | 0.674 | 0.003 | 0.003 | 0.225 |
